# Supplementary material for: A real-world clinical validation for AI-based MRI monitoring in multiple sclerosis
Source: NPJ Digit Med. 2023 Oct 19;6:196. doi: 10.1038/s41746-023-00940-6 (PMC10587188; doi:10.1038/s41746-023-00940-6)
Supplement: Supplementary file 1 — SUPPLEMENTARY FILES [file 41746_2023_940_MOESM1_ESM.pdf]

SUPPLEMENTARY Methods

Core lab quality criteria for assessment of scan suitability for cross sectional image analyses

The core lab standard operating procedure (SOP) for observational studies was implemented in the current work. Images were excluded from cross-sectional analysis volumetric analysis if:

1. Acquisition problems, based on visual inspection, are present in scan, namely:

(a) the brain was not scanned from the base of the brain stem to the vertex.
(b) inadequate tissue contrast, spatial contrast or image inhomogeneity that is likely to compromise analysis quality
(c) images contain artifacts such as aliasing, Gibbs or Truncation, Zipper, Motion or Susceptibility artifacts that are likely to compromise analysis quality

2. Image slice thickness, image geometry and detailed parameter settings are study-specific and specified by the core lab external to the SOP. For the current study, gapless 3DT1 and 3DFLAIR (for lesion in-painting) sequences with a thickness of ≤3mm were specified, but no specific parameter settings were pre-specified.

Determination of longitudinal scanner/protocol stability for lesion and brain volumetric analyses

iQ-MS

Longitudinal inconsistency between timepoints is determined by any of:

1. Scanner mismatch: scans are acquired on a different scanner at the two timepoints

2. Protocol mismatch: scans at the two timepoints are not acquired with similar protocols. Similar protocols are defined as protocols in which the Acquisition Voxel Size does not change by more than 30%. The Acquisition Voxel Size is calculated by [Row Pixel Spacing] * [Column Pixel Spacing] * [Number of Rows] * [Number of Columns] * [Slice Thickness] * [Non-zero elements in the Acquisition Matrix] where all referenced quantities are extracted from the DICOM headers.

Additionally, follow-up and baseline images must meet the following criteria (specified in and automatically extracted from the DICOM headers):

3D-T1

- Same MRAcquisitionType
- Same DeviceSerialNumber
- Difference in EchoTime <= 2
- Difference in RepetitionTime <= 2
- Difference in InversionTime <= 50
- Difference in FlipAngle <= 0.01
- Difference in NumberOfAverages <= 0.01

3D-FLAIR

- Same MRAcquisitionType
- Same DeviceSerialNumber
- Difference in EchoTime <= 10
- Difference in RepetitionTime <= 50
- Difference in InversionTime <= 50
- Difference in FlipAngle <= 0.01
- Difference in EchoTrainLength <= 4
- Difference in NumberOfAverages <= 0.01

3. Affine similarity mismatch: scans at the two timepoints have affine similarity <0.2, implemented as described previously [1]

Core Lab

The core lab standard operating procedure (SOP) for observational studies was implemented in the current work. Relevant elements of the SOP are included below.

Longitudinal inconsistency between timepoints is determined by any of:

1. Scanner mismatch: scans are acquired on a different scanner at the two timepoints

2. Acquisition problems, based on visual inspection, are present in scans at either timepoint, namely:

   (d) the brain was not scanned from the base of the brain stem to the vertex.
   (e) inadequate tissue contrast, spatial contrast or image inhomogeneity that is likely to compromise analysis quality
   (f) images contain artifacts such as aliasing, Gibbs or Truncation, Zipper, Motion or Susceptibility artifacts that are likely to compromise analysis quality

3. Protocol (image geometry and detailed parameter settings) inconsistency: these metrics are study-specific and specified by the core lab external to the SOP. For the current study, no specific parameter deviations were pre-specified.

Supplementary Tables

Supplementary Table 1. Demographic, disease and treatment data for 282 unique patients. [1]Age at most recent included scan ; [2]Subtype at time of most recent study scan. One patient with RRMS was subsequently re-classified as MOG-antibody disease; [3]includes treatments used for at least 6 months during the inter-scan epoch; and immune-reconstitution therapies (alemtuzumab, cladribine) used previously provided no other immunotherapy since; [4]classed as high-efficacy therapy

Mean Age (yrs)[1]		46.0 (21.1-75.9)
Sex		F:M=198:84
MS Subtype[2]	RIS	3
	CIS	1
	RRMS	258
	SPMS	17
	PPMS	3
Disease Duration (years)		13.1 (0.71-41.83)
EDSS		1.5 (0.0-7.0)
Primary Treatment during inter-scan epoch[3]	Beta-interferon	9
	Glatiramer acetate	16
	Teriflunomide	4
	Dimethyl fumarate	35
	Fingolimod	38
	Cladribine	10
	Ocrelizumab[4]	52
	Ofatumumab[4]	4
	Natalizumab[4]	57
	Alemtuzumab[4]	11
	Siponimod	9
	Other	1
	Nil	36

Supplementary Figures

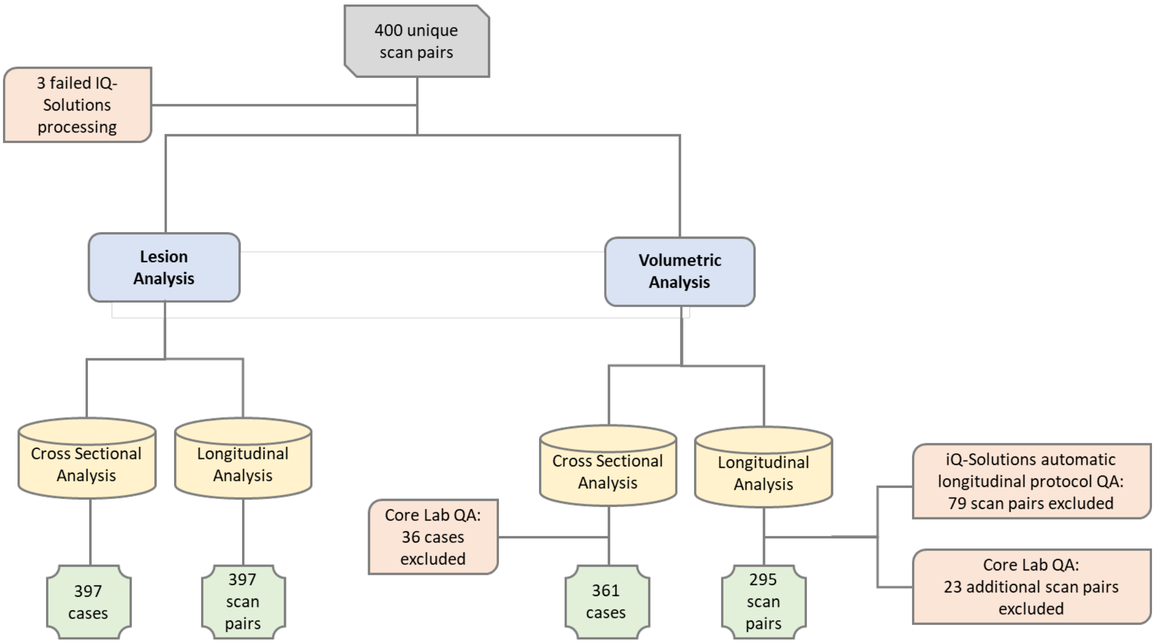

Supplementary Figure 1. Scan inclusion workflow. Exclusion of cases/scan pairs (pink boxes) based on quality criteria described in the supplementary data.

Supplementary References

1. Sima D. M., Horáková D., Nguyen A.-L., Van Hecke W., Kalincik T., Barnett M. H., et al. (2019). Assessing the reliability of longitudinal MRI examinations in multiple sclerosis follow-up. ECTRIMS Online Libr. 278907:547.
