# Supplementary material for: A real-world clinical validation for AI-based MRI monitoring in multiple sclerosis
Source: NPJ Digit Med. 2023 Oct 19;6:196. doi: 10.1038/s41746-023-00940-6 (PMC10587188; doi:10.1038/s41746-023-00940-6)
Supplement: Supplementary file 2 — Reporting Summary [file 41746_2023_940_MOESM2_ESM.pdf]

nature portfolio

Corresponding author(s): Chenyu Wang

Last updated by author(s): Aug 27, 2023

Reporting Summary

Nature Portfolio wishes to improve the reproducibility of the work that we publish. This form provides structure for consistency and transparency in reporting. For further information on Nature Portfolio policies, see our Editorial Policies and the Editorial Policy Checklist.

Statistics

For all statistical analyses, confirm that the following items are present in the figure legend, table legend, main text, or Methods section.

n/a	Confirmed	
☐	☒	The exact sample size (*n*) for each experimental group/condition, given as a discrete number and unit of measurement
☐	☒	A statement on whether measurements were taken from distinct samples or whether the same sample was measured repeatedly
☐	☒	The statistical test(s) used AND whether they are one- or two-sided<br>*Only common tests should be described solely by name; describe more complex techniques in the Methods section.*
☐	☒	A description of all covariates tested
☐	☒	A description of any assumptions or corrections, such as tests of normality and adjustment for multiple comparisons
☐	☒	A full description of the statistical parameters including central tendency (e.g. means) or other basic estimates (e.g. regression coefficient) AND variation (e.g. standard deviation) or associated estimates of uncertainty (e.g. confidence intervals)
☒	☐	For null hypothesis testing, the test statistic (e.g. *F*, *t*, *r*) with confidence intervals, effect sizes, degrees of freedom and *P* value noted<br>*Give P values as exact values whenever suitable.*
☒	☐	For Bayesian analysis, information on the choice of priors and Markov chain Monte Carlo settings
☒	☐	For hierarchical and complex designs, identification of the appropriate level for tests and full reporting of outcomes
☐	☒	Estimates of effect sizes (e.g. Cohen's *d*, Pearson's *r*), indicating how they were calculated

Our web collection on statistics for biologists contains articles on many of the points above.

Software and code

Policy information about availability of computer code

Data collection	The datasets analysed in the current study are available from the corresponding author on reasonable request and with a relevant research agreement.
Data analysis	The underlying code for iQ-SolutionsTM is not publicly available for proprietary reasons; however, code for specific individual algorithms is described in the relevant references [17-19]

For manuscripts utilizing custom algorithms or software that are central to the research but not yet described in published literature, software must be made available to editors and reviewers. We strongly encourage code deposition in a community repository (e.g. GitHub). See the Nature Portfolio guidelines for submitting code & software for further information.

Data

Policy information about availability of data

All manuscripts must include a data availability statement. This statement should provide the following information, where applicable:

- Accession codes, unique identifiers, or web links for publicly available datasets
- A description of any restrictions on data availability
- For clinical datasets or third party data, please ensure that the statement adheres to our policy

The datasets analysed in the current study are available from the corresponding author on reasonable request and with a relevant research agreement.

Research involving human participants, their data, or biological material

Policy information about studies with human participants or human data. See also policy information about sex, gender (identity/presentation), and sexual orientation and race, ethnicity and racism.

Reporting on sex and gender	Our findings apply to both sexes (Male and Female); Sex was considered in the study design as a covariance; Sex was determined based on self-reporting; Sex data were provided in the Supplementary table 1; Sex info was collected during subject's clinic visit; Male: Female = 198: 84.
Reporting on race, ethnicity, or other socially relevant groupings	Not relevant to this study.
Population characteristics	Mean Age : 46.0 (21.1-75.9)
Recruitment	Patients with a diagnosis of MS attending the Royal Prince Alfred Hospital MS Service were retrospectively included in the study.
Ethics oversight	The University of Sydney Human Research Ethics Committee

Note that full information on the approval of the study protocol must also be provided in the manuscript.

Field-specific reporting

Please select the one below that is the best fit for your research. If you are not sure, read the appropriate sections before making your selection.

☒ Life sciences ☐ Behavioural & social sciences ☐ Ecological, evolutionary & environmental sciences

For a reference copy of the document with all sections, see nature.com/documents/nr-reporting-summary-flat.pdf

Life sciences study design

All studies must disclose on these points even when the disclosure is negative.

Sample size	Based on a significance level of 5%, assumed 80% sensitivity of radiologist reports for detection of MS lesion activity, and power of 80% to identify a 10% improvement with iQ-MS, recruitment to the study ended when 400 appropriate scan pairs had been included.
Data exclusions	Please refer to subplementary Figure 1. Scan inclusion workflow. Exclusion of cases/scan pairs (pink boxes) based on quality criteria described in the supplementary data.
Replication	IQ-Solutions analysis are fully automated and reproducible method. Core MRI reading facility are ISO-9001 certified and CFR-21 Part 11-compliant, using standard operating procedures (SOP) designed for regulatory MS clinical trials and the outcome is reproducible.
Randomization	Not applicable. The grouping is defined by methods used.
Blinding	The investigators were blinded during data collection and analysis: The data were collected retrospectively with only following imaging criteria: a minimum of two available MRI timepoints, separated by at least 6 months. Scans with 3D T1-w and 3D FLAIR imaging, acquired on any MRI scanner, were included in the study; there were no pre-specified sequence parameters. All clinical radiology report then subsequently extracted retrospectively. The Core MRI reading and IQ-Solutions MS report were performed independently at the end with only MRI scans available (no patient information were presented).

Reporting for specific materials, systems and methods

We require information from authors about some types of materials, experimental systems and methods used in many studies. Here, indicate whether each material, system or method listed is relevant to your study. If you are not sure if a list item applies to your research, read the appropriate section before selecting a response.

Materials & experimental systems

n/a	Involved in the study	
☒	☐	Antibodies
☒	☐	Eukaryotic cell lines
☒	☐	Palaeontology and archaeology
☒	☐	Animals and other organisms
☒	☐	Clinical data
☒	☐	Dual use research of concern
☒	☐	Plants

Methods

n/a	Involved in the study	
☒	☐	ChIP-seq
☒	☐	Flow cytometry
☐	☒	MRI-based neuroimaging

Magnetic resonance imaging

Experimental design

Design type: Retrospective MRI analysis

Design specifications: Inclusion criteria included a minimum of two available MRI timepoints, separated by at least 6 months. Scans with 3D T1-w and 3D FLAIR imaging, acquired on any MRI scanner, were included in the study; there were no pre-specified sequence parameters

Behavioral performance measures: Not applicable

Acquisition

Imaging type(s): Structural

Field strength: 3 T

Sequence & imaging parameters: Scans with 3D T1-w and 3D FLAIR imaging, acquired on any MRI scanner, were included in the study; there were no pre-specified sequence parameters

Area of acquisition: Whole Brain

Diffusion MRI: ☐ Used ☒ Not used

Preprocessing

Preprocessing software: IQ-MS v1.9.0 for Brain extraction; N4 Bias field correction (ANTS)

Normalization: the whole brain volume and thalamus volume reported in this study are normalized by multiply a subject specific scaling factor. The scaling factor were derived from the linear co-registration of subject's skull and MNI template Skull segmentation masks.

Normalization template: MNI152

Noise and artifact removal: Not applicable

Volume censoring: Not applicable

Statistical modeling & inference

Model type and settings: N/A

Effect(s) tested: N/A

Specify type of analysis: ☒ Whole brain ☐ ROI-based ☐ Both

Statistic type for inference (See Eklund et al. 2016): N/A

Correction: N/A

Models & analysis

n/a	Involved in the study
☒	☐ Functional and/or effective connectivity
☒	☐ Graph analysis
☒	☐ Multivariate modeling or predictive analysis
